# Prevalence and risk factors of HTLV-1/2 and other blood borne infectious diseases among blood donors in Yaounde Central Hospital, Cameroon

**DOI:** 10.11604/pamj.2018.30.125.14802

**Published:** 2018-06-13

**Authors:** Michel Kengne, Dorine Carol Wouado Tsata, Thérèse Ndomgue, Julius Mbekem Nwobegahay

**Affiliations:** 1 Department of Medical Microbiology and Immunology, School of Health Sciences-Catholic University of Central Africa, Yaoundé Cameroon; 2 Assistant Laboratory Technicians School, Yaoundé, Cameroon; 3 Military Health Research Center (CRESAR), Yaounde, Cameroon

**Keywords:** Prevalence, risk factors, HTLV-1/2, HBV, HCV, HIV, syphilis, blood donors

## Abstract

**Introduction:**

Transfusion-transmissible infectious microorganisms including bacteria and viruses are among the greatest threats to blood safety for the recipient. The prevalence and risk factors of HTLV-1/2 and other blood borne infectious diseases were determined among blood donors in Yaounde Central Hospital, Cameroon.

**Methods:**

Design: cross sectional study. Setting: The blood bank unit of Yaounde Central Hospital, Cameroon. Subjects: a consecutive sample of 265 apparently healthy adult blood donors. Investigations: Search for the presence of hepatitis B surface antigen (AgHBs) and antibodies to human T-lymphotropic virus type 1 (anti-HTLV-1/2), human immunodeficiency virus (anti-HIV), hepatitis C virus (anti-HCV) and syphilis and to determine the epidemiological correlates, if any, in the occurrence of HTLV infection.

**Results:**

77 (29.05%) of the blood donors had serological evidence of infection with at least one pathogen and 4 (5.2%) had dual infections with HTLV-1/2. The overall prevalence of HTLV-1/2, HIV, HCV, HBV and syphilis were 5.7%, 5.3%, 2.6%, 11.7%, 3.8% respectively. Surgical history (Chi^2^=4.785; P=0.029), scarification (Chi^2^=6.359; P = 0.012), piercing (Chi^2^ = 16.353; P = 0.000) and intravenous drug use (Chi^2^ = 15.660; P = 0.000) were identified as risk factors for HTLV-1/2 infection.

**Conclusion:**

A relative high prevalence of viral infections and syphilis was recorded among the study participants especially for HTLV-1/2 for which none blood donation is routine screened in our set up. Therefore, a routine screen of blood prior to transfusion should include anti-HTLV-1/2 tests.

## Introduction

The risk of viral and bacterial infections constitutes the major cause of transfusion related morbidity and mortality. Although the risk of transfusion-transmitted infections today is lower than ever due to the current efforts and strategies that have greatly helped reduce transfusion-associated risks [1], the supply of safe blood products remains subject to contamination with known and yet to be identified human pathogens [2, 3]. According to UNAIDS (1997), up to 4 million blood donations worldwide are not tested each year for human immunodeficiency virus (HIV) and few are tested for hepatitis B and C viruses [4]. In sub-Saharan countries, blood safety remains an issue of major concern in transfusion medicine where national blood transfusion services and policies, appropriate infrastructure, trained personnel and financial resources are inadequate. Data on blood donors screening have revealed a high prevalence for the presence of viral and bacterial markers indicative of HBV, HIV, HCV and syphilis infections [5]. Similarly, those infections have been reported to be prevalent in Cameroon [6] and the screening of blood donors for HB surface antigen (HBsAg), HCV, HIV and syphilis antibodies is henceforth a routine practice in the blood banks [7].

Although a priority is given to screen blood for antigens and antibodies of the cited infectious agents in our set up, continuous monitoring of the magnitude of transfusion-transmissible infections among blood donors is important for assessing the occurrence of infections in the blood donor's population and for estimating the epidemiology of the diseases in the community. Aside from HIV, HCV, HBV and syphilis, a number of other infectious agents transmitted by transfusion of blood products have been described. Human T cell lymphotropic viruses I is (HTLV-I) associated with adult T cell leukemia and HTLV-associated myelopathy/ tropical spastic paraparesis [8]. Both retroviruses (HTLV-1/2) have been attributed a role in the increased risk for developing severe asthma, respiratory and urinary tract infections, uveitis and dermatitis [9-12]. The global epidemiology of HTLV varies widely with prevalence rates up to 10% in certain areas in Japan, and up to 5-6 % in countries of the Caribbean, the Sub-Saharan Africa, and areas in the Middle East and South America [12]. In our set up, no priority has ever been given to screen blood donors for antibodies to HTLV-1/2 [7] thereby making blood transfusion unsafe for blood recipients. Thus, evaluation of data on the prevalence of these transfusion transmitted infectious agents among blood donors is important for estimating the risk of transfusion and optimizing donor recruitment strategies to minimize infectious diseases transmission due to these viruses namely HTLV-1/2. Therefore, this study was conducted to determine among blood donors at the Yaounde Central Hospital in Cameroon, the seroprevalence of HTLV, HIV, HCV, HBsAg, and syphilis and to determine the epidemiological correlates, if any, in the occurrence of HTLV infection.

## Methods

This was a cross sectional and prospective study which was carried out in the blood bank unit of the Yaounde Central Hospital in Cameroon between June and November 2014. The hospital is a tertiary level hospital that provides health services to over twenty thousand inhabitants in the capital city, Yaounde. A consecutive sample of 265 apparently healthy adult blood donors agreed to participate after an informed consent. Participants were either volunteers or family donors who were recruited by patients, their families or friends to replace blood used or expected to be used for patients from the blood bank of the hospital. Confidentiality was ensured at all stages of the study. A unique identification code was assigned to each participant. A designed questionnaire was used to collect data from individuals over a panel of questions on the past history of blood transfusion, sexual behavior and practice, medical and sociodemographic histories. For each study subject, five milliliters of blood was collected and sera from all the blood donors were then checked for the presence of hepatitis B surface antigen (HBsAg) and the antibodies to HIV-1/2, HCV and syphilis using the automated system i1000SR immunoassay (abbot) following the manufacturer's instructions. Similarly, antibodies to HTLV-1/2 were detected using ELISA technique (HTLV-1/2 ELISA 4.0, CE MARK, MP Biomedicals, France) according to the manufacturer's instructions. ABO and Rh blood groups determinations were carried out on a slide using monoclonal blood grouping antisera; anti-A, anti-B, anti-AB and anti-D (BIOTEC Laboratories Ltd, Great Britain) following the manufacturer's instructions.

**Data analysis**: Data were entered and analyzed using SPSS version 16.0 for windows (SPSS, Inc, Chicago, IL). Discrete variables were compared using the Chi-square test. Statistical significance difference was considered at value of p<0.05.

**Ethics**: A written informed consent was obtained from the study subjects prior to enrollment. Permission to conduct the study was obtained from the Director of the Yaoundé Central Hospital; while ethical clearance was obtained from the institutional ethics committee of the School of Health Sciences, Catholic University of Central Africa (N°2015/006/CEIRSH/ESS/MIM).

## Results

As shown in Table 1, a total of 265 consecutive apparently healthy adult blood donors were screened at the blood bank unit of the Yaoundé Central hospital during the study period. Donors were aged between 18 and 52 years (mean 28±6.6 years) and 184 (69.4%) were in the age group of 18-30 years. Of these, 242 (91.30%) were males compared to 23 (8.7 %°) females (sex ratio M/F = 10:1), 159 (60%) were workers, 114 (43.0%) had a secondary level of education, 189 (71.3%) lived in urban areas, 222 (83.8%) were unmarried, 117 (44.2%) were first time donors, 235 (88.7%) were voluntary donors, 140 (52.8%) were blood group O and 252 (95.0%) were Rhesus D positive. 77 (29.05%) of the blood donors had serological evidence of infection with at least one pathogen and 4 (5.2%) had dual infections with HTLV-1/2. The overall prevalence of HTLV-1/2, HIV, HCV, HBV and syphilis were 5.7%, 5.3%, 2.6%, 11.7%, 3.8% respectively (Table 2). The differences in the prevalence of HTLV-1/2 between the age group, sex, profession, educational level, residence, marital status, donor status, type of donors and rhesus/ABO blood group of the donors were not statistically significant (P > 0.05) (Table 1). Statistically significant association was observed between HTLV-1/2 infection and the following factors such as surgical history of the blood donors (Chi^2^=4.785; P=0.029), scarification (Chi^2^=6.359; P=0.012), piercing (Chi^2^=16.353; P=0.000) and intravenous drug use (Chi^2^=15.660; P=0.000) (Table 3).

**Table 1:** Sociodemographic characteristics of the study subjects and correlates with HTLV-1/2 infection

Characteristics	Frequency (%) (total donors=265)	No. of HTLV-1/2 positive cases (%)	Statistics
Age groups (years)			
18-30	184 (69.4)	09 (4.8)	Chi^2^= 0.669 P= 0.71
31-40	67 (25.3)	05 (7.4)	
> 40	14 (5.3)	01 (7.1)	
**Gender**			
Male	242 (91.7)	15 (6.1)	Chi^2^= 1.512 P= 0.219
Female	23 (8.7)	0 (0.0)	
**Profession**			
Unemployed	106 (40)	09 (8.4)	Chi^2^= 2.650 P= 0.104
Worker	159 (60)	06 (3.7)	
**Educational level**			
Primary	61 (23)	06 (9.8)	Chi^2^= 2.588 P= 0.274
Secondary	114 (43)	05 (4.3)	
Higher	90 (34)	04 (4.4)	
**Residence**			
Rural	76 (28.7)	03 (3.9)	Chi^2^= 0.586 P= 0.440
Urban	189 (71.3)	12 (6.3)	
**Marital status**			
Single	222 (83.8)	12 (5.4)	Chi^2^= 0.167 P= 0.683
Married	43 (16.2)	3 (6.9)	
**Type of donors**			
Family	235 (88.7)	12 (5.1)	Chi^2^= 1.193 P= 0.275
Volontary	30 (11.3)	03 (10.0)	
**Donors status**			
First time donation	117 (44,2)	3 (2.5)	Chi^2^= 3.761 P= 0.052
Repeat donation	148 (55.8)	12 (8.1)	
**ABO/Rhesus blood group**			
A+	64 (24.1)	5 (7.8)	Chi^2^= 2.112 P= 0.909
A-	1 (0.3)	0 (0.0)	
AB+	11 (4.1)	0 (0.0)	
B+	45 (16.9)	3 (6.6)	
B-	4 (1.5)	0 (0.0)	
O+	132 (49.8)	7 (5.3)	
O-	8 (3.0)	0 (0.0)	

**Table 2:** Prevalence of blood borne pathogens among blood donors

Pathogens	Prevalence (%)
VIH	14 (5.3)
HBV	31 (11.7)
HCV	7 (2.6)
Syphilis	10 (3.8)
HTLV-1/2	15 (5.7)
Total	77 (29.05)
HTLV-1/2 and HIV	2 (2.6)
HTLV-1/2 and HBV	1 (1.3)
HTLV-1/2 and HCV	0 (0)
HTLV-1/2 and Syphilis	1 (1.3)
Total	4 (5.2)

**Table 3:** Factors associated with HTLV-1/2 infection

Factors	Frequency (%) (total donors=265)	No. of HTLV-1/2 positive cases (%)	Statistics
Surgical history			
yes	9 (3.4)	2 (22.2)	Chi^2^= 4.785 P= 0.029
no	256 (96.6)	13 (5.0)	
**Scarification**			
yes	33 (12.4)	05 (15.1)	Chi^2^= 6.359 P= 0.0012
no	232 (87.6)	10 (4.3)	
**Piercing**			
yes	19 (7.2)	05 (26.3)	Chi^2^= 16.353 P= 0.000
no	246 (92.8)	10 (4.06)	
**Intravenous drug use**			
yes	08 (3.1)	03 (37.5)	Chi^2^= 15.660 P= 0.000
no	257 (96.9)	12 (4.6)	

## Discussion

This study aimed at determining the prevalence and risk factors of HTLV-1/2 and other blood borne infectious diseases among blood donors in the Yaounde Central Hospital of Cameroon. According to WHO recommendations, the minimum requirement for all donor blood should include screening for HIV, HBV and syphilis [6]. A national strategy of infectious disease screening of donated blood is well implemented in the blood bank unit of the Yaounde Central Hospital in Cameroon and includes the testing for HIV, HBV, HCV, and T. pallidium. Screening for HTLV-1/2 is not routine in this center thus the anti HTLV-1/2 test that was used in this study was purchased. In our study, the overall prevalence of HTLV-1/2, HIV, HCV, HBV and syphilis were 5.7%, 5.3%, 2.6%, 11.7%, 3.8% respectively. This result is in agreement with those reported in Africa [6, 7, 13,14]. The prevalence of HBV was remarkedly high among the study population. It has been extensively reported a very large burden of HBsAg infection in all sub-Saharan African regions with carrier rates ranging from 9 to 20% [14]. Our finding may be due to the low hepatitis B vaccine coverage rates in the study set up. This study illustrates the current transfusion transmissible risk of the screened infectious agents in Cameroon and raises serious concerns regarding the safety of the blood supply in our community, even after the screening of donors for infectious agents. There is still a potential risk of transmitting viral infections during the serologic window period i.e. the period of early infectivity when an immunologic test is non-reactive [15]. Therefore, the absence of infectious agents in the blood of apparently healthy individuals may not be sufficient to ensure lack of circulating pathogens.

In a study conducted in India by Bhattacharya et al [16], it was observed that out of the 1027 HBsAg negative blood samples screened, 18.3% were found to be anti-HBc positive and 21.3% of the anti-HBc positive samples were HBV DNA positive by PCR. This figure should serve as a remainder to health personnel to take the necessary precautions, including reducing the number of unnecessary transfusions that will help to lower the risk of the most frequent transfusion transmitted infections to blood recipients. To explore the risk factors for HTLV-1/2 seropositivity, we examined sociodemographic and lifestyle variables among blood donors. It was found that HTLV-1/2 infection was not associated to age, sex, profession, educational level, residence, marital status, donor status, and type of donors. The same trend wasn't observed in other studies. For example, in studies carried out by Douardo et al [17] and Santos et al [18], age was found to be a risk factor for HTLV-1 infection. A potential explanation for this association was that increasing age provides a greater length of exposure to events that might result in acquiring the virus, such as sexual activity, intravenous drug use or blood transfusion [18]. The difference in the size of the study population may explain the discrepancy with our result. In this study, we observed that individuals with surgical history, scarification, piercing and who used intravenous drug are at higher risk for HTLV-1/2 infection. This result is in agreement with the finding of Blattner [19] who demonstrated that HTLV-1 is transmitted by routes involving transfusion and needle sharing.

## Conclusion

This study illustrates the current transfusion transmissible risk of HTLV-1/2, in the study set up. The data also reveal that individuals with surgical history, scarification, piercing and who used intravenous drug are at higher risk for HTLV-1/2 infection. From these observations, we recommend routine screening of blood donor for HTLV-1/2 antibodies.

### What is known about this topic

- HTLV-1/2, HBV, HIV, HCV and syphilis are the infectious agents transmitted by transfusion of blood products;
- The screening of blood donors for HB surface antigen (HBsAg), HCV, HIV and syphilis antibodies is a routine practice in the blood banks in Cameroon except for HTLV-1/2;
- Several individual behaviors and exposures are associated with HTLV-I seropositivity, corresponding to the known modes of transmission: from mother to child, predominantly through breastfeeding; via sexual intercourse and via parenteral transmission by transfusion of infected cellular blood products or sharing of needles and syringes.

### What this study adds

- The overall prevalence of HTLV-1/2 is 5.7% in the study set up;
- Surgical history of the blood donors, scarification, piercing and intravenous drug use are associated with HTLV-1/2 infections.

## Competing interests

The authors declare no competing interests.
